# Effects of biological therapies on patients with Type-2 high asthma and comorbid obesity

**DOI:** 10.3389/fphar.2023.1315540

**Published:** 2024-01-08

**Authors:** Diya Garg, Loretta G. Que, Jennifer L. Ingram

**Affiliations:** ^1^ Department of Pathology and Laboratory Medicine, Neurology, and Biological Chemistry, Irvine, CA, United States; ^2^ Division of Pulmonary, Allergy, and Critical Care Medicine, Duke University Medical Center, Durham, NC, United States

**Keywords:** asthma, obesity, Type 2 inflammation, biomarkers, biological therapy

## Abstract

Over 20 million adults and 6 million children in the United States (US) have asthma, a chronic respiratory disease characterized by airway inflammation, bronchoconstriction, and mucus hypersecretion. Obesity, another highly prevalent disease in the US, is a major risk factor for asthma and a significant cause of diminished asthma control, increased submucosal eosinophilia, and reduced quality of life. A large subgroup of these patients experiences severe symptoms and recurrent exacerbations despite maximal dosage of standard asthma therapies. In the past two decades, the development of biological therapies has revolutionized the field and advanced our understanding of type 2 inflammatory biomarkers. However, patients with obesity and comorbid asthma are not principally considered in clinical trials of biologics. Large landmark cluster analyses of patients with asthma have consistently identified specific asthma phenotypes that associate with obesity but may be differentiated by age of asthma onset and inflammatory cell profiles in sputum. These patterns suggest that biologic processes driving asthma pathology are heterogenous among patients with obesity. The biological mechanisms driving pathology in patients with asthma and comorbid obesity are not well understood and likely multifactorial. Future research needs to be done to elicit the cellular and metabolic functions in the relationship of obesity and asthma to yield the best treatment options for this multiplex condition. In this review, we explore the key features of type 2 inflammation in asthma and discuss the effectiveness, safety profile, and research gaps regarding the currently approved biological therapies in asthma patients with obesity.

## 1 Introduction

Asthma is a common chronic respiratory disease affecting 262 million children and adults worldwide in 2019, with high mortality in low and lower-middle-income countries (Global Initiative For Chronic Obstructive Lung Disease, 2023). Asthma is a heterogeneous noncommunicable disorder characterized by airway inflammation, airway hyperresponsiveness (AHR), bronchoconstriction, wheezing, and mucus hypersecretion (Haldar et al., 2008). It manifests with respiratory symptoms and airflow obstruction that vary in severity and impact on quality of life. Metabolic and cardiovascular conditions, especially obesity, diabetes mellitus, and atrial fibrillation contribute to the heterogeneity of severe asthma (Boulet and Boulay, 2011). Moreover, nearly 60% of patients with severe asthma are experiencing obesity, complicating the diagnosis, pathobiology, and treatment of their asthma (Peters et al., 2018).

While most patients with asthma see improvements in their symptoms through inhaled corticosteroids (ICSs) and long-acting beta2-agonists (LABA), adults and children experiencing obesity have increased recurrent exacerbations despite maximum dose treatment (Henderson et al., 2020). These patients demonstrate glucocorticoid resistance and poor asthma control with increased asthma severity, consuming a disproportionately high amount of healthcare resources for their asthma management (Henderson et al., 2020). These patients are defined as having severe asthma and are under consideration for alternative therapies. Alternative therapies focusing on controlling severe asthma target pathobiological mechanisms contributing to asthma severity and responsiveness to usual therapies (Ernst and Posadzki, 2012). Several pathways have gained attention, but specifically, the use of monoclonal antibodies to target components of Type-2-high (T2-high) airway inflammation have emerged as effective therapies for a subset of asthma patients. The T2-high endotype describes the degree (high) of type 2 lymphocytes (CD4^+^), innate lymphoid cells group 2 (ILC2s) and type 2 cytokines (interleukin [IL]-4, IL-5, and IL-13) that drive airway inflammation through increasing eosinophils, basophils, and mast cells in the airways (Moore et al., 2010; Kuruvilla et al., 2019a; Kaur and Chupp, 2019). Over the last 25 years, biological therapies have revolutionized severe asthma by targeting T2-high severe asthma to reduce asthma exacerbations, but little research has evaluated the efficacy of these therapies in patients with asthma and comorbid obesity.

Given that both asthma and obesity are on the rise, and these patients are relatively unresponsive to standard medications, clinicians are investigating alternative medications such as biological agents. Thus, understanding the effectiveness of using biological agents in patients with asthma with comorbid obesity would impact asthma management in this challenging patient population. This review is aimed to examine and explain the effectiveness of approved biological agents in people with asthma with comorbid obesity and provide an overview to help clinicians select medications to manage T2 severe asthma phenotypes exhibiting comorbid obesity.

## 2 Pathobiology of severe asthma

Our understanding of T2-high asthma has matured over the last two decades, with key findings for biomarkers representing this phenotype: bronchoalveolar lavage (BAL) fluid, blood and sputum eosinophilia, high levels of serum immunoglobulin-E (IgE), and high fractional exhaled nitric oxide (FeNO) measurements (Dunican and Fahy, 2015a; Fahy, 2015a). Patients presenting with inflammation defined by T2-high immune responses comprise 50%–70% of the severe asthma patient population (AllergyAsthma Network, 2023). In these patients, the airway is characterized by chronic eosinophilic inflammation, excessive T2 cytokine (IL-4, IL-5, and IL-13) release, increased activation of T-helper 2 cells (Th2) and ILC2s, and pathologic interplay of neutrophils, basophils, lymphocytes, dendritic cells, and mast cells (Fahy, 2015a; Gandhi et al., 2016; Busse et al., 2021).

Severe asthma is a heterogenous disease, and recent efforts to classify phenotypes of severe asthma have focused on T2 inflammatory markers as defining characteristics. Allergen-sensitized asthma is typically characterized by early age of asthma onset (<12 years of age) and the production of elevated allergen-specific IgE, with the majority of patients being responsive to corticosteroid therapy (Walford and Doherty, 2014). This phenotype causes more severe disease, AHR and eosinophilia through Th2 cell and ILC2 activation, and T2 cytokine secretion (Romanet-Manent et al., 2002). Non-allergic/T2-low sensitized asthma is typically characterized by late age of asthma onset (≥12 years of age) that does not display elevated serum IgE levels (Hurwitz, 1955). T2-low asthma is more arbitrarily defined as asthma without features of T2 cytokine-driven inflammation. Acute severe exacerbations are less responsive to corticosteroids in the non-allergic phenotype (Klain et al., 2022). T2-low asthma has been reported to comprise about 20% of the severe asthma patient population and occurs more frequently in late-onset females with obesity (Ricciardolo et al., 2021a). It is associated with Th1 and Th17 cell-driven, neutrophilic airway inflammation, and elevated quantities of cytokines IL-1β, IL-6, IL-8, IL-17, tumor necrosis factor-alpha (TNF-α), and interferon-gamma (IFN-γ) involved in its pathobiology (Hinks et al., 2021; Ji and Li, 2023). T2-low asthma is difficult to define due to the lack of signature biomarkers and is primarily diagnosed based on the absence of or very reduced levels of eosinophils and other T2-driven inflammation markers and relatively high levels of neutrophils in sputum (Rupani et al., 2021). However, recent reports of longitudinal real-world severe asthma cohorts demonstrate that the T2t1-low asthma phenotype may be rare compared to the T2-high phenotype, as the vast majority of severe asthma patients exhibited elevated eosinophilic inflammation and other T2 biomarkers at times over a 10-year period (Azim et al., 2021; Heaney et al., 2021; Rupani et al., 2023). Thus, patients described as T2-low may actually have an underlying T2-high phenotype that is masked at the time of sampling, and the simplified T2-high/T2-low paradigm should be expanded to include other cellular/molecular pathways to define asthma phenotypes.

Infections, bacteria, smoking, and atrial fibrillation can provoke severe non-allergic asthma (Ricciardolo et al., 2021b). Patients with T2-low asthma, compared to the T2-high asthma phenotype, do not respond as well to current standard treatments for asthma, such as ICS, LABAs, and higher-level treatments such as biologics (Ricciardolo et al., 2021b). Knowledge of the various and distinct asthma pathobiology phenotypes is continuously growing; however, the underlying mechanisms of severe asthma pathogenesis are not yet completely understood. Further research is needed to understand the cellular and metabolic processes of all phenotypes of severe asthma and to find specific interventions to best treat patients according to their specific endotypes.

**FIGURE 1:**
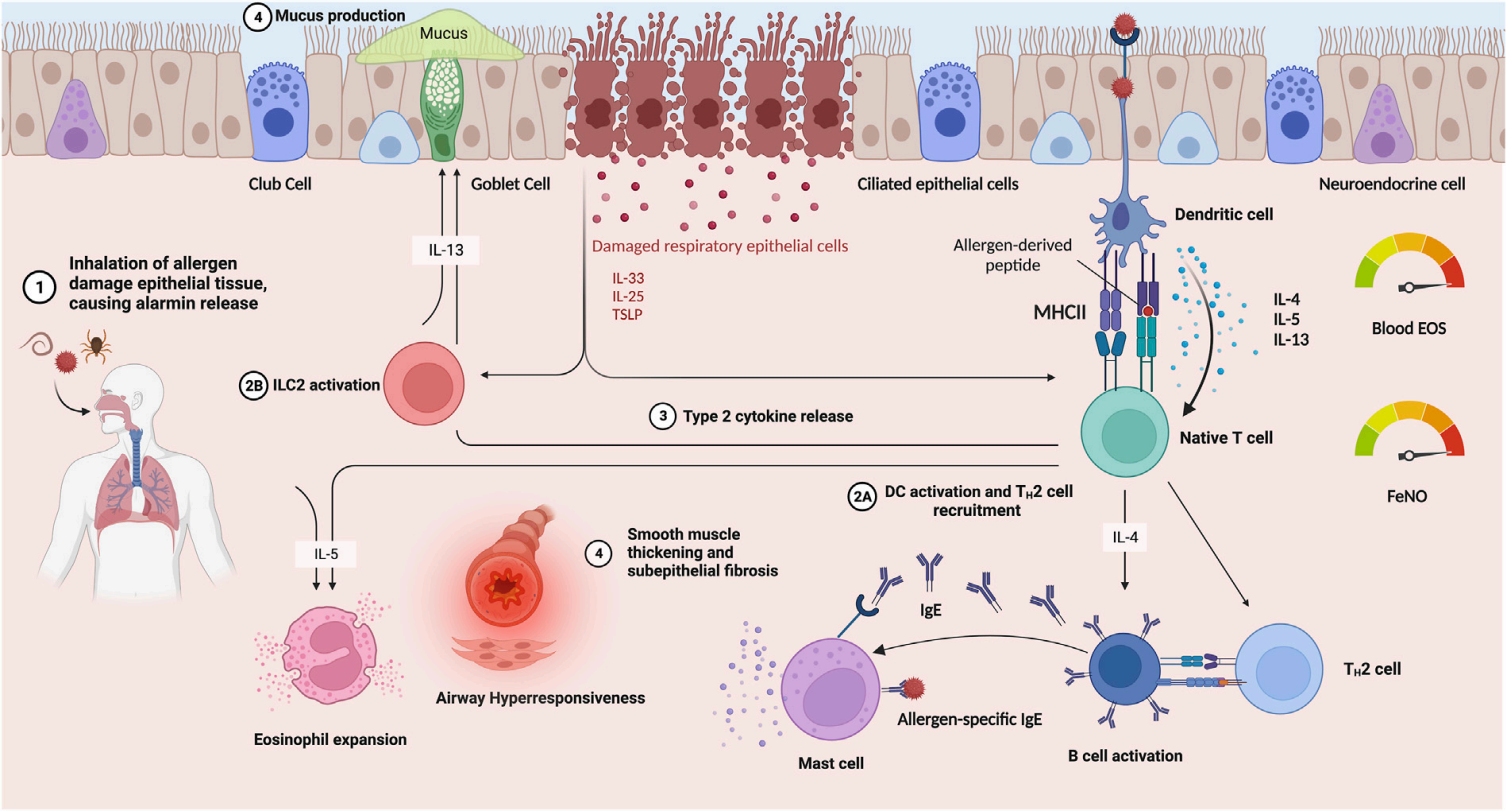
T2-driven asthma immune responses occur when an inhaled allergen (1) injures airway epithelium, leading to alarmin (IL-33, IL-25 and TSLP) release and in parallel, stimulates dendritic cell activation, leading to T2 cell recruitment and release of T2 cytokines (IL-4, IL-5 and IL-13) (2A, 2B). Alarmin signaling activates ILC2s (2) and release of T2 cytokines (3). T2 cytokine signaling stimulates eosinophil and mast cell recruitment and activation, as well as class-switching of B cells to secrete IgE. These responses lead to goblet cell metaplasia, increased mucus production, bronchoconstriction and AHR, and airway remodeling (4). A commonly used biomarker to identify T2 asthma is through elevated blood eosinophil count. Additionally, in allergic inflammation, nitric oxide (NO) is produced by the airway epithelium in excess, so elevated FeNO also provides a reliable index of T2 inflammation. Created with BioRender.com Abbreviations: TSLP, thymic stromal lymphopoietin; DC, dendritic cells; EOS, eosinophils; IL, interleukin; IgE, Immunoglobulin E; T2, Type-2 helper; MHC II, Major Histocompatibility Complex 2; FeNO, fractional concentration of exhaled nitric oxide.

T2 immune responses in the airway are mediated primarily by eosinophils, dendritic cells, mast cells, Th2 cells, ILC2s, and IgE-producing B-cells (Koyasu and Moro, 2011; Walker and McKenzie, 2018). After exposure to an allergen, the T2 immune response is initiated upstream by alarmins, IL-33, IL-25, and thymic stromal lymphopoietin (TSLP) (Koyasu and Moro, 2011; Walker and McKenzie, 2018). They stimulate migration of ILC2s to the airway epithelium and subepithelial mucosa and regulate the differentiation of native Th cells (Th0) into Th2 cells (Barnes, 2001). ILC2s also secrete Type 2 cytokines (IL-4, IL-5, and IL-13) that promote AHR and mucus overproduction (Table 1) (Walker and McKenzie, 2018; Akdis et al., 2020). These T2 cytokines drive a cascade of downstream events, including activating airway epithelial cells, switching B cell-secreted IgG to IgE, and inducing exaggerated responses to inhaled agents, causing remodeling changes in the airway (Fahy, 2015a). The relevant pathological remodeling changes are smooth muscle hypertrophy, goblet cell metaplasia, and subepithelial fibrosis (Dunican and Fahy, 2015a). The distinct functions of T2 cytokines and the interrelationship between asthma exacerbations and airway remodeling in T2-high asthma are of considerable interest and clinical relevance (Figure 1).

**TABLE 1:** The central drivers of T2-high asthma are IL-4, IL-5, and IL-13 that drive airway inflammation through increasing eosinophils, basophils, and mast cells in the airways. These distinct cytokines have unique functions contributing to the pathobiology of asthma.

Central drivers of T2-high asthma		
Mediator	Impact on asthma pathobiology	References
IL-4	T2 cell differentiation, B cell class-switching, airway remodeling	[Dunican and Fahy (2015a), Steinke and Borish (2001)]
IL-5	Regulator of eosinophil proliferation, migration, activation and survival	[Pelaia et al. (2019a), Greenfeder et al. (2001)]
IL-13	Goblet cell hyperplasia, mucus hyperproduction, B cell class-switching, airway remodeling	[Marone et al. (2019), Rael and Lockey. (2011)]

## 3 Asthma and obesity

The incidence of obesity has tripled in the US in the past 40 years, and body mass indices (BMIs) have nearly doubled from the pre-pandemic rate (Office of the Surgeon General US, 2010). Nearly 70% of adults exhibit either overweight or obesity, with more than 30% of those having obesity (NHLBI Obesity Education Initiative Expert, 1998; National Institute of Diabetes and Digestive and Kidney Diseases, 2021; USAFacts, 2023). Obesity is defined by a BMI greater than 30 kg/m^2^ and is typically characterized by excessive adiposity (Nuttall, 2015). Despite the reliance of this clinical definition of obesity on BMI, the measure has serious limitations for describing body composition, which is influenced not only by adipose tissue, lean muscle and bone mass and does not capture the heterogeneity of obesity phenotypes, including differences in body composition (Bray, 2023). Waist circumference is a more reflective measure of central adiposity than BMI, and body fat distribution may be more accurately measured using dual x-ray absorptiometry (DXA), computed tomography (CT) or magnetic resonance imaging (MRI) (Bray, 2023). Still, BMI provides a standard measure by which to track populations of patients and to serve as a base indicator of individual metabolic health (Bray, 2023).

Obesity often exists alongside asthma and within the context of other comorbidities (gastroesophageal reflux disease [GERD], metabolic syndrome, diabetes, cardiovascular disease, obstructive sleep apnea, anxiety and depression, etc.,.) (Pandya et al., 2014). These concurrent conditions may affect long-term clinical presentation of asthma and influence the effect of asthma-focused biologic drug delivery and efficacy. Evidence from both human clinical trials and mouse models of asthma demonstrate bidirectionality of various comorbidities in altering systemic and airway immune responses, including those targeted by biologic therapies (Thomas et al., 2010; Broytman et al., 2015; Tariq et al., 2019; Fernández-Gallego et al., 2022; Wang et al., 2022; Hou et al., 2023). For example, the presence of GERD reduced airway production of several T2 cytokines in a mouse model allergic airway disease and markedly altered airway production of proteins involved in inflammatory responses in patients with severe asthma (Thomas et al., 2010; Tariq et al., 2019). Moreover, chronic intermittent hypoxia, as experienced in obstructive sleep apnea, reduced airway levels of IL-5 and increased Type1 inflammation (Broytman et al., 2015; Ohta et al., 2020). Also, multimorbidity may lead to misdiagnosis, undertreatment or overtreatment of asthma. A recent study found that 36% of patients with obesity were misdiagnosed with asthma by physicians (Scott et al., 2012). In addition, comorbid conditions, including obesity, may lead to polypharmacy with negative effects on efficacy of long-term treatments, such as biologics, by reducing adherence and increasing risk of adverse events (Ye et al., 2022). Thus, the complex interplay of the pathobiology of asthma, obesity and other comorbid conditions that impact immune responses must be considered in aggregate when developing diagnoses and therapeutic strategies.

Indeed, certain asthma treatments increase the risk of developing comorbid conditions, including obesity. Children and adolescents with asthma and comorbid obesity are 24% more likely to be unresponsive to bronchodilators, even when given systemically (Peters et al., 2018). Despite poor response, they are often treated with these drugs for long periods of time, only worsening obesity and metabolic dysfunction. Oral corticosteroids (OCS) are commonly prescribed for severe asthma, but patients on these drugs are at significantly increased risk for weight gain, among other adverse effects (Price et al., 2018; Kulkarni et al., 2022). Initiation of biologic therapies reduces the use of systemic corticosteroids (Chen et al., 2023). Moreover, long-term weight reduction was associated with biologic therapy in patients with severe eosinophilic asthma, an effect that was attributed to reduced OCS use. The greatest weight loss was observed in patients that had the highest exposure to OCS before initiating biologic therapy combined with a reduction in OCS dose during treatment (Ten Have et al., 2023). Therefore, biologic therapies could be particularly beneficial for OCS-treated asthma patients with obesity in mitigating both comorbid conditions.

**FIGURE 2:**
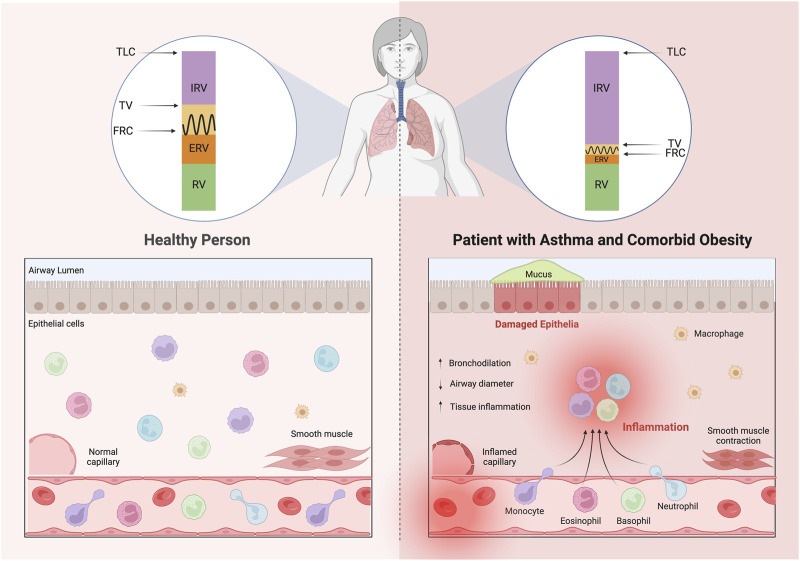
Patients with asthma and comorbid obesity exhibit altered lung volumes causing rapid, shallow breathing patterns due in part to physical restriction of the lungs by excess adipose tissue, but also due to reduced airway diameter, a consequence of thickened airway walls and loss of airway elasticity. Increased airway recruitment of inflammatory cells promotes airway narrowing through stimulation of mucus hypersecretion, smooth muscle layer thickening and subepithelial fibrosis. Abbreviations: TLC, total lung capacity; IRV, inspiratory reserve volume; TV, tidal volume; FRC, functional residual capacity; ERV, expiratory reserve volume; RV, residual volume; AHR, airway hyperresponsiveness.

Asthma is more severe in patients with obesity than in lean patients (Sharma and Cowan, 2021). Patients with asthma and comorbid obesity may be classified in two endotypes according to age of asthma onset: one with early-onset asthma (<12 years of age) with obesity as a complicating factor or even a consequence of asthma and the other with late-onset asthma (≥12 years of age), which is associated with the development of obesity prior to the onset of asthma (Holguin et al., 2011; Sideleva and Dixon, 2014). While mechanisms of asthma pathobiology in patients with comorbid obesity are not fully understood, it may be attributed to altered cell signaling and inflammatory mechanisms inherent in obesity. The manifestation of airway remodeling could be provoked by an abnormal accumulation of T2 cytokine–producing cells and eosinophils in visceral adipose tissue (Figure 2).

The mechanical properties of the lungs and chest are significantly altered in obesity. The mechanisms behind the reduction in the respiratory drive in obesity and asthma have not been clearly elucidated. Obesity causes adipocytes to undergo hypertrophy, resulting in inadequate perfusion of enlarged adipose tissue, hypoxia and apoptosis (Kang et al., 2023; Luk et al., 2023). Various researchers have studied obesity and its relation with lung volumes and found that obesity causes an accumulation of fat in the thoracic and abdominal cavities which is associated with increased pleural pressures secondary to restricted downward movement of the diaphragm and outward movement of the chest wall (Ladosky et al., 2001; Jones and Nzekwu, 2006; Melo et al., 2014; Dixon and Peters, 2018). This fat accumulation alters the breathing pattern resulting in a substantial reduction of functional residual capacity (FRC); the volume remaining in the lungs after a normal, passive exhalation; expiratory reserve volume (ERV); the volume of air that can be forcefully exhaled after a normal resting expiration, and tidal volume (TV); the volume of air that moves in and out of the lungs with each respiration (Ladosky et al., 2001; Jones and Nzekwu, 2006; Melo et al., 2014; Dixon and Peters, 2018). Obesity has very little effect on residual volume (RV); the volume of air remaining in the lungs after maximum forceful expiration; and total lung capacity (TLC); the volume of air in the lungs upon the maximum effort of inspiration (Ladosky et al., 2001; Jones and Nzekwu, 2006; Melo et al., 2014; Dixon and Peters, 2018). Obesity is proposed to cause a substantial increase of inspiratory reserve volume (IRV), the amount of air that can forcefully be inhaled after a normal volume, because of increased workload of the intercostal muscles (Figure 2) (Ladosky et al., 2001; Jones and Nzekwu, 2006; Melo et al., 2014; Dixon and Peters, 2018).

Recent studies have shown increased numbers of proinflammatory macrophages as well as elevated concentrations of the pro-fibrotic adipokine, leptin, TNF-α, and IL-6 in bronchoalveolar lavage fluid (BALF) and blood from adolescents and adults with severe asthma (Stream and Sutherland, 2012; Sutherland et al., 2012; Farzan, 2013), contributing to disease severity and diminished responsiveness to prescribed therapies. Obesity causes rapid adipose tissue expansion, leading to the activation of hypoxia-inducible factor 1-alpha (HIF1-α) (He et al., 2011). Hypoxic death of adipocytes promotes fibrosis and proliferation of M1 macrophages that induce pro-inflammatory activity (Weisberg et al., 2003). The number of adipose macrophages in non-obese patients is 4% but can reach up to 12% in patients with obesity (Weisberg et al., 2003). With the elevation of adipose M1 macrophages, pro-inflammatory cytokines are activated and attract fibroblasts and more pro-inflammatory cells (Boutens and Stienstra, 2016). Notably, Th17 lymphocytes are also elevated in adipose tissue, which release IL-6 and IL-17, inducing the polarization of T cells into Th17 cells and reducing secretion of the anti-inflammatory adipokine, adiponectin (Boutens and Stienstra, 2016). Adiponectin suppresses differentiation and activation of M1 macrophages, contributing to a decreased inflammatory process (Boutens and Stienstra, 2016). Little is known regarding the influence of these obesity-related inflammatory pathways on the efficacy of asthma biologics and whether measurement of serum IL-6, IL-17 or adiponectin levels may predict response to biologic therapies in patients with asthma. These gaps in knowledge represent important areas of future research.

Asthma severity in patients with obesity may be directly addressed through weight loss. Studies have shown that a 5% decrease in body weight can reduce airway inflammation (Johnson et al., 2022). However, obesity is increasing—by 2030, 51% of the US population is expected to exhibit obesity, a significant jump from the 38% population who have obesity in 2022 (Schneider et al., 2010; Finkelstein et al., 2012). In 2018, a higher proportion of adults with obesity had asthma (39%) than did not have asthma (27%) (Centers for Disease Control and Prevention, 2013). Weight loss through dietary modification and exercise is difficult. Bariatric surgery and pharmacologic interventions of patients with asthma result in improvement asthma control; however, long-term consequences are poorly understood, and these approaches must be accompanied by positive lifestyle management strategies to maximize benefits for asthma patients (Reddy et al., 2011). Dietary interventions carry the lowest risk of adverse events and are the core strategy to tackle obesity in a sustainable manner. Therefore, further understanding the interaction between asthma and obesity and its direct treatment options to improve asthma control is increasingly important.

## 4 Biological therapies in Type-2 high asthma

T2-high asthma constitutes 60% of patients with severe asthma (AllergyAsthma Network, 2023). Patients with severe uncontrolled asthma have increased hospitalizations, poor quality of life, and impaired lifestyles compared to patients with controlled asthma (Johnson et al., 2022). Although T2-high asthma is generally a corticosteroid-responsive endotype, a substantial proportion of patients with asthma and comorbid obesity (44%) have persistent symptoms despite more than a year of ICS and LABA usage (Tashiro and Shore, 2019). Although ICS remains essential for managing acute exacerbations, the overuse of systemic corticosteroids is associated with adverse effects, including osteoporosis, immunosuppression, obesity, sleep apnea, arrhythmias, glaucoma, and depression (Yasir et al., 2023). Therefore, patients receiving the standard of care for severe asthma may remain poorly controlled.

However, over the past decade, this unmet need to improve severe asthma outcomes has led to a greater understanding of our knowledge of the complex pathophysiological mechanisms of severe asthma, leading to the development of new treatment options. Today, patients with uncontrolled severe T2-high asthma are routinely considered candidates for biologics, monoclonal antibody-based cytokine-targeted therapies (Votto et al., 2021). Unlike corticosteroids which target an unspecified range of cells, biological therapies specifically target inflammatory cytokines (IL-4, IL-5, and IL-13), of T2-high asthma (Votto et al., 2021). Biologics have the potential to modify the natural course of the disease without the collateral damages associated with ICS and LABA (Dragonieri and Carpagnano, 2021). While ICS inhibits IL-13, a high proportion of uncontrolled asthma patients have elevated levels of IL-13 in their sputum despite treatment with high dosage ICS (Saha et al., 2008). The impact of biological therapies on asthma control and corticosteroid dependence has led researchers to consider asthma remission as a possibility. Recent evidence has identified a new phenotype, super-responders, for patients taking biologic treatments. Super responders are defined by a group of patients with a more rapid response to a certain treatment leading to better asthma control and quality of life (Portacci et al., 2023). The super responder rate is estimated to be between 14% and 44% and more commonly is observed in patients with male sex, no smoking history, a lower BMI, higher T2 biomarker levels, and a later age of disease onset (Jane McDowell et al., 2023; Portacci et al., 2023). Obesity influences potential for asthma remission on biological therapies, with a 47% lower frequency of remission in patients with obesity compared to patients without obesity (Jane McDowell et al., 2023).

As mentioned above, biologic therapies influence long-term (2-year) weight loss through sparing of OCS, which contributes to weight gain as a side effect (Ten Have et al., 2023). Significant weight loss following initiation of anti-IL-5 therapies in the short-term (6–11 months) has been reported, but these findings were not replicated in a separate study (Kuruvilla et al., 2019b; Cusack et al., 2021). Regardless, a subgroup of asthma patients has been identified that gained a significant amount of weight at the short- or long-term time points in the studies (Kuruvilla et al., 2019b; Cusack et al., 2021; Ten Have et al., 2023). The factors contributing to this weight gain in the subgroup are likely complex and postulated to be due to depletion of eosinophils by anti-IL-5-targeted biologics, influencing adipose tissue homeostasis and body weight (Calco et al., 2020). More research is needed to understand the mechanisms driving weight gain and loss in association with biologics in asthma with comorbid obesity.

Biological therapies were first introduced with an anti-IgE monoclonal antibody, omalizumab, and now have subsequent biological agents aimed at different inflammatory modulators, including IL-4 receptor alpha (IL-4Rα), IL-5, IL-5 receptor alpha (IL-5Rα), IL-13, and TSLP (Pelaia et al., 2018a; Guntern and Eggel, 2020). These drugs block specific immunological pathways triggering allergic airway inflammation (Pelaia et al., 2018a; Guntern and Eggel, 2020). To date, the United States Food and Drug Administration (FDA) has approved six biologics for use in selected severe asthma patients: Omalizumab (anti-IgE); Mepolizumab, Benralizumab, Reslizumab (anti-IL5/anti-IL5R ***α***); Dupilumab (anti-IL4R ***α***); and Tezepelumab (anti-TSLP) (Guntern and Eggel, 2020).

### 4.1 Anti-IgE

The role of immunoglobulin E (IgE) is well-established in allergic asthma. During an allergen exposure, IgE antibodies bind to dendritic cells, mast cells, and basophils through high-affinity IgE receptors (FcεRIα) are cross-linked with allergens, triggering release of proinflammatory mediators such as IL-4 (Chipps and Marshik, 2004; Owen, 2007).

Omalizumab (Xolair^®^) is a recombinant, humanized monoclonal antibody that binds to IgE and prevents it from cross-linking with the IgE receptor and downregulating FcεRI-mediated production by basophils and mast cells (Chapman et al., 2006). Omalizumab prevents asthma exacerbations by blocking the release of inflammatory mediators (histamine and tryptase) from mast cells and reducing the infiltration of eosinophils in the airway (Chapman et al., 2006). Omalizumab was the first targeted biologic therapy developed and licensed for severe allergic asthma (Kumar and Zito, 2023). It was approved by the Food and Drug Administration (FDA) in 2003 for patients >12 years of age and in 2016 for patients >6 years of age with persistent allergic asthma (U.S. Food and Drug Administration, 2014). Several clinical studies have demonstrated that omalizumab reduces the frequency of severe asthma exacerbations in about 60%–70% of severe non-obese BMI asthma patients (Solèr et al., 2001; Owen, 2007; Holgate et al., 2009; Niven et al., 2016; Kotoulas et al., 2022; Xolair, 2023).

Unlike previous data on the effect of omalizumab on lean patients, studies of patients with obesity have contradicting results. In a double-blind, placebo-controlled study, Geng *et al* examined the response to omalizumab based on BMI (Geng et al., 2022). The authors recruited patients aged 12–75 years with severe allergic asthma who were symptomatic despite treatment with high doses of ICS (Geng et al., 2022). They found that omalizumab reduced asthma exacerbations, BDP doses, improved lung function, asthma symptom scores, and asthma-related total symptom score (TSS) across all BMI categories compared with placebo (Geng et al., 2022). However, interestingly, they found patients with obesity on omalizumab had greater reductions of exacerbations than normal patients relative to placebo. The reduction of exacerbations in patients with BMI ≥30 (−86.9%) was higher than in patients with BMI<25 (−37.4%) (Geng et al., 2022). They found no significant differences in forced expiratory volume in 1 s (FEV1) improvement across all subgroups (Geng et al., 2022). Patients with obesity started with worse mean baseline total asthma symptom score (TASS) and AQLQ scores, and after treatment, they had reduced improvement (95% CI) compared to lean patients (Geng et al., 2022). The rate of exacerbations with omalizumab was similarly low between the BMI categories (Geng et al., 2022).

In contrast to the findings of Geng *et al*, other studies have found that omalizumab was less effective in reducing exacerbations in asthma patients with obesity compared to non-obese patients. For example, Sposato *et al* conducted a real-world study (n = 340) and found that patients with obesity had a 3-fold higher risk of exacerbations (odds ratio, 3.114; CI 95%, *p* = 0.002) compared with non-obese patients after more than 1 year of omalizumab treatment.^102^ Furthermore, obesity was associated with reduced FEV1 (*β* = −6.981, *p* = 0.04), FVC (*β* = −11.689, *p* = 0.014) and Asthma Control Test (ACT) scores (*β* = −2.585, *p* = 0.027) and was associated with a higher FENO level (*β* = 49.045, *p* = 0.040) than non-obese patients (Sposato et al., 2018).

The findings of Geng *et al* which demonstrated an improvement in exacerbations in patients with obesity, correlated with a prospective study done by Oliveira et al. (2019) conducted a small (n = 32) study for 12 months and found that the 19 patients with obesity taking omalizumab had a higher reduction in exacerbations (6.0) *versus* non-obese patients (0.4; *p* < .001) (Oliveira et al., 2019). At the end of the study, patients with obesity had significantly better lung function (FEV1) than non-obese patients (70.2% vs. 58.8%, *p* = 0.017) (Oliveira et al., 2019). In another study, Gibson *et al* demonstrated that the response of severe asthma patients treated with omalizumab was similar regardless of obesity status (Gibson et al., 2016). However, the above-mentioned studies lacked testing with a placebo, which possibly allowed Geng *et al* to observe a higher exacerbation rate with increasing BMI.

Evidence shows that omalizumab reduces asthma exacerbations in patients despite their BMI. Clearly, omalizumab reduces exacerbations in non-obese patients from age six or older; however, it is unclear how this therapy biologically affects patients with obesity. Published studies present varied data, possibly because the study either did not have a placebo control or had a small sample size for the numbers of patients with *versus* without obesity (Table 2). Therefore, these experiments need to be replicated on a large scale to determine a definitive effect of omalizumab in patients with obesity.

**TABLE 2:** Main findings of studies comparing BMI on omalizumab response in severe asthma patients.

Characteristic	Geng *et al* Geng et al., (2022)	Sposato *et al* Sposato et al., (2018)	Oliveira *et al* Oliveira et al., (2019)	Gibson *et al* Gibson et al., (2016)
**Study Design**	Data from two phase III studies. Randomized, double-blind, placebo-controlled studies	Real world retrospective study conducted in Italy. No placebo used	Non-interventional prospective study conducted in an outpatient asthma clinic in Portugal. Comparative analysis over 12 months	Registry design. Used a non-interventional, observational database of omalizumab therapy. Clinicians in over 21 clinics in Australia enrolled patients from October 2011 and June 2014
**Requirements**	Patients had to be on ICS dosages equivalent to 420–840 µg per day or 500–1,200 µg per day of BDP for 3 months before randomization	Patients had to be on systemic corticosteroids for at least 3 days prior and/or hospitalized in the past	Patients ensured their diet and physical activity remained the same during the study. Equivalent doses of budesonide were given	Participants had moderate incompletely reversible airflow limitation with bronchodilators
**Age (yrs)**	18–75	44–63	Mean age = 53; no range specified	12–85; mean age = 51.4
**Sample Size**	BMI <25, n = 397; (placebo, n = 194; omalizumab, n = 203)	BMI <25, n = 117	BMI <25, n = 13	BMI <25, n = 99
	BMI >25 - <30, n = 330 (placebo, n = 169; omalizumab, n = 161)	BMI >25 - <30, n = 146		
			BMI ≥30, n = 19	BMI ≥30, n = 81
	BMI ≥30, n = 268 (placebo, n = 128; omalizumab, n = 140)	BMI ≥30, n = 77		
**Treatment**	During run-in, all patients were switched to inhaled BDP and then tapered off. After the run-in period, patients were randomized to receive either omalizumab or placebo subcutaneously at 0.016 mg/kg body weight per IU of total serum IgE/mL every 2 or 4 weeks based on their body weight and total IgE at screening	Daily dosage of BDP was low (≤500 mg), medium (500–1,000 mg) or high (≥1,000 mg), according to GINA classification of ICS dose equivalence. All patients also received a monthly omalizumab dose of 450 mg	Patients received omalizumab at 2- or 4- week intervals based on the serum IgE levels and body weight as recommended. They also had a daily dosage of BDP (1,200 μg)	Patients received omalizumab at 2- or 4- week intervals. Non-obese received 450 mg, while patients with obesity received 600 mg
**Duration**	4- to 6-week run-in period, a 16-week corticosteroid-stable phase, and a 12-week corticosteroid-reduction phase	32 months	12 months	6 months
**Effect on Corticosteroid dosage**	Lower levels in patients with obesity taking omalizumab (600 vs. 504 μg). Lower levels in patients with obesity compared to non-obese patients (672 μg)	Increased level in patients with obesity compared to non-obese patients (OR:4.448)	Not tested	Similar in patients with obesity and non-obese patients
**Effect on FEV1 reversibility**	**↑**	Reduced response compared to non-obese BMI (*β* = −6.981)	**↑↑**	**↑**
	Slight FEV1 increase in patients with obesity taking omalizumab (2080 mL vs. 2,410 mL). Lower levels in patients with obesity than non-obese patients (2,140 mL)		Higher in patients with obesity (70.2%) than non-obese patients (58.8%)	0.6% increase in patients with obesity taking omalizumab (68.8%–69.4%)
**Effect on blood eosinophils (cells/μL)**	**↓**	Not tested	Not tested	Not tested
	Slight decrease in patients with obesity taking omalizumab (245 vs. 240)			
**Effect on FeNO level**	Not tested	Increased FENO level in patients with obesity (*β* = 49.045)	Not tested	Not tested
**Effect on serum total IgE**	**↑**	Not tested	Not tested	**↓↓**
	Increase in patients with obesity taking omalizumab (121.0 vs. 142.0). Marked decrease in non-obese BMI (181.5 vs. 156.0)			Lower levels in patients with obesity (268) compared to non-obese patients (306)
**Effect on asthma exacerbations**	**↓↓**	3-fold higher risk of exacerbations in patients with obesity than in those with non-obese BMI (OR: 3.114)	**↓↓**	Not tested
	−72% decrease of exacerbation in patients with obesity taking omalizumab. Only −37.4% decrease in patients with non-obese BMI.		−85% decrease in patients with obesity	
**Asthma Control**	Slight improvement of asthma control. AQLQ scores for patients with obesity taking omalizumab (4.2 vs. 4.0). No improvement in patients with non-obese BMI. Slight improvement in TASS scores for patients with obesity taking omalizumab (4.1 vs. 4.0). No improvement in patients with non-obese BMI.	Slight improvement of asthma control. ACT scores for patients with obesity taking omalizumab (*β* = −2.585)	Major improvement of asthma control. ACT scores increased from 10.6 to 23.3 in patients with obesity	Slight improvement of asthma control. AQLQ scores decreased by 1.4 points in patients with obesity over the course of 12 months (T0 = 3.6, T 12 = 2.2)
**Limitations**	Post-hoc analyses	Lack of placebo-controlled study. Lack of data on key T2 high biomarkers	75% patients were female; lack of placebo-controlled study; small number of patients; lack of data on key T2 high biomarkers	63% were female; lack of placebo-controlled study; lack of data on key T2 high biomarkers

Abbreviations: BDP, beclomethasone dipropionate; ICS, inhaled corticosteroids; LABA, long-acting beta agonists; BMI, body mass index; IgE, Immunoglobulin E; ACT, Asthma Control Test that ranges from 5 (poor control of asthma) to 25 (complete control of asthma); ACLQ, asthma control questionnaire with scores ranging from 0 (totally controlled) and 6 (severely uncontrolled); TASS, total asthma symptom score that ranges from 0 (totally controlled) and 5 (severely uncontrolled); FEV1, forced expiratory volume.

### 4.2 Anti-IL-5 and Anti-IL5Rα

Interleukin (IL)-5 is central in initiating the eosinophilic airway inflammation associated with severe asthma. IL-5 binds to the alpha chain of its specific receptor (IL5Rα), regulating the development, migration, and survival of eosinophils (Pelaia et al., 2019a). Eosinophils are pleiotropic, multifunctional leukocytes facilitating an innate response against inhaled allergens, modulating an inflammation cascade in airway (Pelaia et al., 2019a). Upon IL-5 activation, eosinophils release cytokines that induce damage to airway epithelial cells and tissues (Greenfeder et al., 2001). Targeting IL-5 or IL-5Rα, the main mediators of eosinophilic inflammation, through monoclonal antibodies can reduce eosinophilia in severe asthma patients with uncontrolled symptoms.

The currently available IL-5/IL-5R-targeting biologics for severe eosinophilic asthma include mepolizumab and reslizumab, two anti-IL-5 antibodies, and benralizumab, an anti- IL-5Rα antibody (Principe et al., 2021). Mepolizumab (Nucala^®^) is a recombinant, humanized monoclonal antibody against IL-5 (IgG1) (Nucala, 2023). This biologic binds to IL-5 and prevents its interaction with the α subunit of the IL-5 receptor (IL-5Rα) (Emma et al., 2018). By blocking the binding of IL-5 to its receptor, mepolizumab selectively inhibits eosinophilic activation, thereby reducing airway inflammation (Emma et al., 2018). The FDA approved mepolizumab as a treatment for severe refractory eosinophilic asthma (>150 cells/µL) in 2015 for patients (≥12 years of age) with uncontrolled asthma undergoing previous treatment (Nucala, 2023). The standard dose is 100 mg, administered every 4 weeks subcutaneously (Nucala, 2023). Reslizumab (Cinqair^®^) is a recombinant humanized monoclonal antibody (IgG4) that, like mepolizumab, targets IL-5 to prevent its binding with IL-5Rα (Padilla Galo et al., 2018; Cinqair, 2023). Reslizumab was approved by the FDA in 2016 as a treatment for patients (≥18 years of age) with severe eosinophilic asthma uncontrolled despite maximum doses of ICS and additional controllers (Padilla Galo et al., 2018; Cinqair, 2023). This biologic is administered intravenously (IV) every 4 weeks (3 mg/kg) to treat patients with peripheral blood eosinophils of ≥400 cells/µl and ≥3 asthma exacerbations in the past 12 months (Padilla Galo et al., 2018; Cinqair, 2023).

**FIGURE 3:**
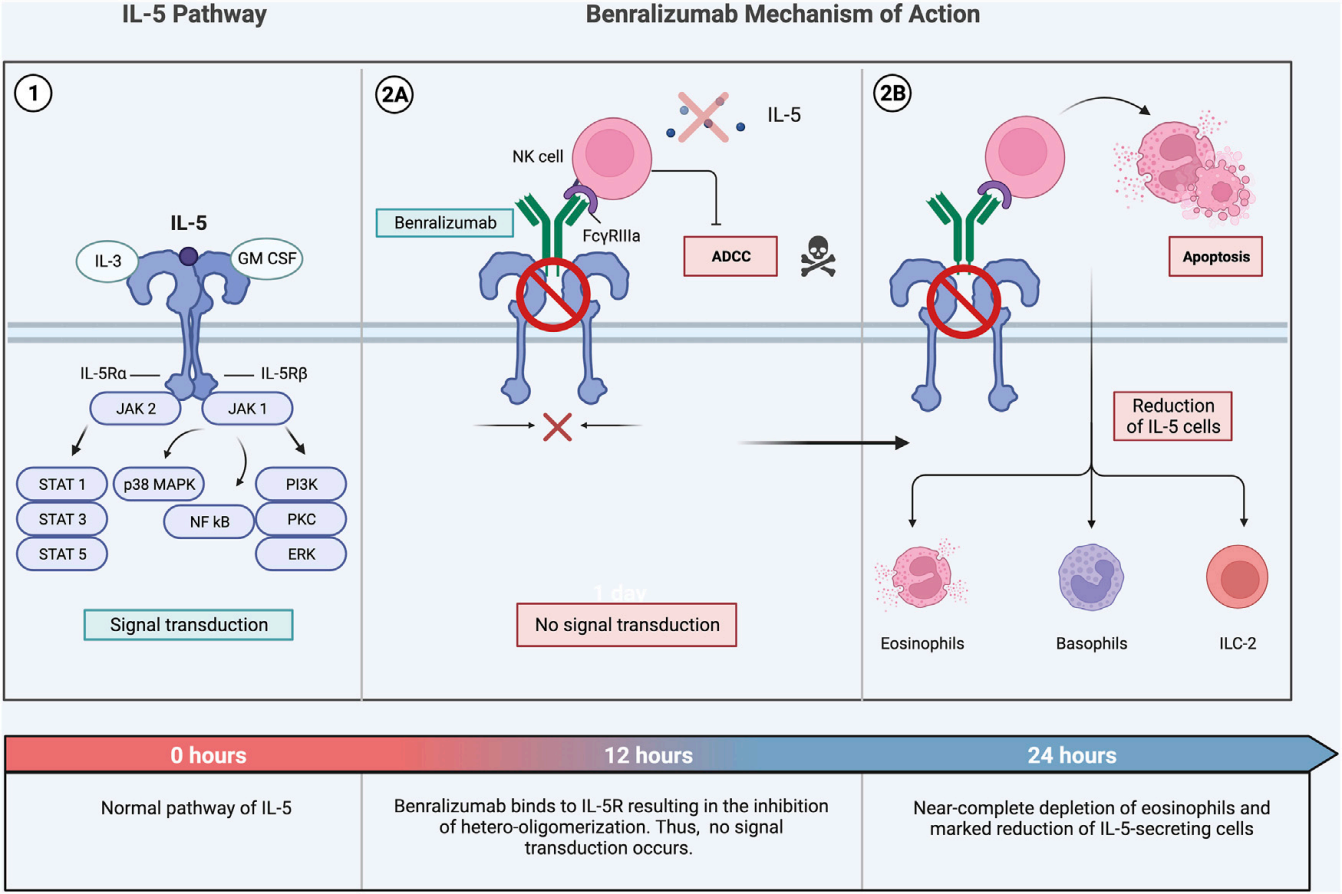
Benralizumab is a humanized monoclonal antibody that targets IL-5Rα (1) (Pelaia et al., 2018b). This biological therapy is unique; it is characterized by a dual mechanism of action (2A, 2B). Through interacting with Fab fragments, benralizumab specifically binds IL-5Rα, inhibiting the interaction between IL-5 and its receptor stopping signal transduction on target cells (2A). Additionally, it interacts with the FcγIIIRa receptor expressed by NK cells triggering ADCC-induced apoptosis of eosinophils through the release of pro-apoptotic proteins such as granzymes. (2B) (Pelaia et al., 2018b) Multiple research studies have found a marked depletion in eosinophil, basophils, and proliferation of B-cells in a span of 24 hours following administration of benralizumab (Jackson et al., 2020). Abbreviations: IL-5, interleukin-5; IL-3, interleukin-3; GM-CSF, granulocyte-macrophage colony-stimulating factor; JAK, Janus kinase; IL-5Rα, interleukin-5 receptor alpha; IL-5Rβ, interleukin-5 receptor beta; STAT, signal transducer and activator of transcription; MAPK, mitogen-activated protein kinase; NF-kB, nuclear factor kappa light chain enhancer of activated B cells; PI3, phosphoinositide 3-kinase; PKC, protein kinase C; ERK, extracellular signal-regulated kinase; NK cells, natural killer cells; ADCC, antibody-dependent cellular cytotoxicity.

Benralizumab (Fasenra^®^) is a humanized afucosylated monoclonal antibody (IgG1) that targets IL5Rα on the surface of eosinophils and basophils (Faserna, 2023). Benralizumab works by binding its Fab fragments to IL5Rα, impeding the assembly of the ternary molecular complex (IL-5, IL5Rα, and βc subunits) of the IL-5 receptor (Figure 3) (Pelaia et al., 2018b; Dávila González et al., 2019). Unlike mepolizumab and reslizumab, benralizumab induces eosinophil apoptosis through antibody-dependent cell-mediated cytotoxicity (Pelaia et al., 2018b; Dávila González et al., 2019). This process occurs because the Fc portion of benralizumab interacts with the surface of the FcγRIIIa receptor of natural killer cells, thus triggering apoptosis (Pelaia et al., 2018b; Dávila González et al., 2019). Apoptosis could possibly cause a more profound decrease of circulating airway eosinophils for all patients with asthma. Benralizumab was approved by the FDA in 2019 as a treatment for severe eosinophilic asthmatics (>12) with ≥300 blood eosinophils/µl (Faserna, 2023). This biologic is administered subcutaneously once (30 mg) a week for the first 4 weeks and then once every 8 weeks (Faserna, 2023).

Findings of several studies relating efficacy of IL-5 or IL-5Ralpha-targeted biologics in patients with asthma and comorbid obesity are summarized in Table 3. In a double-blind, placebo-controlled study, Albers *et al* examined the rate of asthma exacerbations in patients taking mepolizumab (Albers et al., 2019). Albers and others conducted a *post hoc* meta-analysis from placebo-controlled, randomized, double-blind trials (Albers et al., 2019). The authors gave the severe asthma patients (n = 936) mepolizumab (100 mg) or placebo every 4 weeks for 24 weeks (Albers et al., 2019). They found that, across BMI categories, mepolizumab treatment resulted in greater reductions from baseline in blood eosinophil count than placebo treatment, with 83% reduction in the ≤25 kg/m^2^ BMI subgroup compared to 76% reduction in the >30 kg/m^2^ BMI subgroup (Albers et al., 2019). However, they found that mepolizumab was less effective in the reduction of exacerbations in patients with obesity than those with non-obese BMIs (Albers et al., 2019). Altered drug bioavailability may explain why patients with obesity display reduced responses to some treatments. Also, across all BMI categories, mepolizumab treatment resulted in an increase from baseline in pre-bronchodilator FEV1 *versus* placebo, with a smaller effect in the highest BMI category (Albers et al., 2019). Interestingly, this analysis showed that varying the amount of the treatment (mepolizumab doses of 75, 100, 250 or 750 mg IV) resulted in similar exacerbation rate reductions across BMI categories with no discernible trend noted in exacerbation reductions with the 250 mg and 750 mg IV doses of mepolizumab, even at the higher weight categories (Albers et al., 2019). Similarly, Fonseca *et al* examined a similar effect in an experiment conducted for 12 months including a total of 25 patients, with 11 patients exhibiting obesity (Da Cunha Fonseca et al., 2022). After administering mepolizumab for the duration of the study, the authors found no statistically significant difference between the two weight groups in any of the variables, except for FVC% predicted, which resulted in a greater improvement in non-obese patients (Da Cunha Fonseca et al., 2022). This study showed a significant reduction in the number of exacerbations in both populations, health-related quality of life, and asthma control independent of obesity (Da Cunha Fonseca et al., 2022). However, non-obese patients had significant lung function improvement compared to patients with obesity, which further reinforces the importance of obesity as a comorbid condition which needs to be intensively addressed in asthma patients (Da Cunha Fonseca et al., 2022).

**TABLE 3:** Main findings of studies comparing BMI on IL-5/IL-5Rα biologic response in severe asthma patients.

Characteristic	Albers *et al* Albers et al., (2019)	Fonseca *et al* Da Cunha Fonseca et al., (2022)	Trudo *et al* Trudo and Martin, (2019)	Menzella *et al* Menzella et al., (2021)	Nanzer *et al* Nanzer et al., (2022)
**Targeted Epitope**	IL-5	IL-5	IL-5Rα	IL-5Rα	IL-5Rα
**Study Design**	Data from two phase III studies. Randomized, double-blind, placebo-controlled studies	Retrospective analysis	Data from two phase III studies	Italian observational retrospective cohort study	Retrospective, observational study in United Kingdom
**Requirements**	Patients with a ≥2 exacerbations with corticosteroids, blood eosinophil count of ≥150 cells/μL or 300 cells/μL in the prior year	Patients with severe eosinophilic asthma	High dosage inhaled corticosteroids or LABA and blood eosinophil count of ≥300 cells/μL		Patients with severe eosinophilic asthma
**Age (yrs)**	≥12	Mean age = 57; no range specified	≥18	≥18; mean age 55.8	≥12
**Sample Size**	BMI <25 kg/m^2^, n = 323; (placebo, n = 157; omalizumab, n = 166)	BMI <25 kg/m^2^, n = 14	n = 986	BMI <25 kg/m^2^, n = 70	BMI <30 kg/m^2^, n = 120
	BMI >25 kg/m^2^ - <30 kg/m^2^, n = 330 (placebo, n = 162; omalizumab, n = 168)			BMI >25 kg/m^2^ - <30 kg/m^2^, n = 79	BMI >30 kg/m^2^ - <40 kg/m^2^, n = 105
		BMI ≥30 kg/m^2^, n = 11		
	BMI ≥30 kg/m^2^, n = 283 (placebo, n = 149; omalizumab, n = 134)			BMI ≥30 kg/m^2^, n = 33	BMI ≥40 kg/m^2^, n = 33
**Treatment**	Patients received Mepolizumab (100 mg) SC or placebo, plus ICS every 4 weeks for either 24 or 32 weeks	Patients received Mepolizumab 100 mg) SC once every 4 weeks for 12 months	Patients received Benralizumab 30 mg SC every 8 weeks (first three doses every 4 weeks) or placebo	Patients received Benralizumab 30 mg SC every 8 weeks (first three doses every 4 weeks)	Patients received Benralizumab 30 mg SC every 8 weeks (first three doses every 4 weeks)
**Duration**	24 or 32 weeks	12 months	48 or 56 weeks	12 months	2 years
**Effect on Corticosteroid dosage**	Not tested	**↓↓**	Not tested	Not tested	**↓↓**
		Less decrease in OCS% used in patients with obesity (4%–2%) than patients with non-obese BMI (7%–1%) through treatment			Less decrease in OCS% used in patients with obesity (59% 33%) than patients with non-obese BMI (64%–28%) through treatment
**Effect on FEV1 reversibility**	43 mL increase in patients with obesity through treatment. 135 mL increase in patients with non-obese BMI through treatment	Significant higher increase in non-obese patients (59%–80%) than patients with obesity (58%–66%)	Greater improvement in non-obese (148 mL) than patients with obesity (214 mL). Baseline values were not provided	Not tested	Not tested
**Effect on blood eosinophils**	**↓↓↓**	**↓↓↓**	Not tested	Not tested	Not tested
	Lower reduction in patients with obesity (76%) than non-obese patients (83%) through treatment	Significant lower levels of patients with obesity through treatment (290 eos/uL to 65eos/uL). Patients with non-obese BMI had a more profound decrease (855 eos/uL to 60 eos/uL)			
**Effect on FeNO level**	Not tested	**↑**	Not tested	Not tested	Not tested
		Higher increase in patients with obesity (20 ppb–45 ppb) than patients with non-obese BMI (40 ppb–50 ppb)			
**Effect on serum total IgE**	Not tested	Not tested	Not tested	Not tested	Not tested
**Effect on asthma exacerbations**	**↓↓**	**↓↓**	Similar reduction of exacerbations in patients with or without obesity. Values were not provided	**↓↓↓**	**↓↓**
	Higher reduction in patients with obesity (51%) than patients with non-obese BMI (38%) with mepolizumab	Higher decrease in patients with obesity (3–0) than in patients with non-obese BMI (2–0)		Similar reduction of exacerbations in patients with (−90.9%) and without (−96%) obesity through treatment	Higher reduction of AER in patients with obesity through treatment (5.7–1.2) than in non-obese patients (4.6–0.9)
	Treatment with mepolizumab doses (75, 250, or 750 mg IV or 100 mg SC) resulted in similar exacerbation rate reductions across all BMIs				
**Asthma Control**	Less improvement in SGRQ score in patients with obesity (−5.7) than in non-obese BMI patients (−7.8) through treatment. Less improvement in ACQ-5 score in patients with obesity (−0.28) than in non-obese BMI patients (−0.46) through treatment	Not tested	Not tested	Lower ACT in patients with obesity (15.5) than lean patients (14.2). Baseline values not provided	Lower improvement ACQ-5 for patients with obesity (3.3–1.9) than lean patients (2.7–1.3)
**Limitations**	Post-hoc analyses	72% patients were female; retrospective analysis	Post-hoc analyses; lack of data on key T2 high biomarkers	61.5% patients were female; lack of data on key T2 high biomarkers	Grouped normal and overweight patients in one cohort

**Abbreviations:** ICS, inhaled corticosteroids; LABA, long-acting beta agonists; IgE, Immunoglobulin E; BMI, body mass index; ACT, Asthma Control Test that ranges from 5 (poor control of asthma) to 25 (complete control of asthma); ACQ-5, asthma control questionnaire with scores ranging from 0 (complete control of asthma) and 6 (severely uncontrolled); FEV1, forced expiratory volume; SEA, severe eosinophilic asthma; OCS, oral corticosteroids; SC, subcutaneously; eos, eosinophil; SGRQ, asthma control test that ranges from 0 (excellent health) and 100 (poor health).

To date, no studies report the stratification of reslizumab efficacy by BMI. In non-obese patients Castro *et al* and various other researchers have demonstrated the administration of reslizumab with a reduction in the rate of asthma exacerbations by 34% (*p* < 0.0001) compared with placebo (Castro et al., 2011). However, cluster studies show that biologics that require weight-based dosing, such as omalizumab and reslizumab, may have less benefit in asthma patients with obesity (Clark and Lebwohl, 2008). This reduction in effectiveness was seen in a recent retrospective review of 340 patients with severe asthma on omalizumab, noting that patients with obesity, compared to those with non-obese weight, demonstrated worse outcomes (Farzan et al., 2022). Specifically, patients with obesity were significantly associated with a greater number of exacerbations, reduced ACT scores, and worse asthma control as defined by the GINA guidelines (Clark and Lebwohl, 2008). Interestingly, a small study (n = 10) conducted by Mukherejee *et al* demonstrated that weight-adjusted IV reslizumab therapy was more effective than fixed-dose mepolizumab therapy in attenuating airway eosinophilia in prednisone-dependent patients with asthma long-term (Mukherjee et al., 2018). The reason for this finding could possibly be due to pharmacokinetics and bioavailability of the biologic. Possibly weight-based dosing may pose benefits in the short term and fixed dosage is beneficial long term.

To examine the effects of benralizumab, Trudo *et al* conducted a *post hoc* analysis on the annual rate of exacerbations of patients with and without obesity taking this biologic (Trudo and Martin, 2019). Trudo and others assessed two Phase III clinical trials, and found that benralizumab decreased asthma exacerbations and increased lung function for patients with severe uncontrolled eosinophilic asthma regardless of BMI (Trudo and Martin, 2019). However, they also found that these improvements were less robust in individuals with obesity than those of non-obese weight (Trudo and Martin, 2019). Menzella *et al* and others compared 182 patients, (n = 70 underweight/lean BMI, n = 70 overweight, and n = 33 with obesity) for 12 months (Menzella et al., 2021). They found that the severe annual exacerbation rate (AER) decreased considerably in all groups with −97.0% in underweight/lean BMI patients, −91.1% in overweight, and −92.9% in patients with obesity (Menzella et al., 2021). While the trend shows that the efficacy of benralizumab diminished with increased BMI, it is interesting to note that patients with obesity had a slightly greater decrease in AER than overweight patients (Menzella et al., 2021). However, these trends need further investigation in larger cohorts to ensure it is representative of this population.

A similar trend is seen in another study with Nanzer *et al* examining the response of long-term benralizumab administration on comorbid obesity in severe eosinophilic asthma (Nanzer et al., 2022). Nanzer and others conducted this study in 258 patients, with 120 patients having BMI <30 kg/m^2^, 105 patients having BMI ≥30–39 kg/m^2^, and 33 patients having BMI ≥40 kg/m^2^ (Nanzer et al., 2022). They found the baseline AER to be 4.6, 5.7, 6.1 ANOVA, *p* = 0.022) and baseline ACQ-6 was 2.7, 3.3, 3.8 (*p* < 0.001) respectively (Nanzer et al., 2022). After 1 year they observed a significant reduction in AER vs. baseline with −78% in non-obese patients, −81% in patients with obesity, and −70% decrease in patients with morbid obesity (Nanzer et al., 2022). This reduction further decreased in the second year, −80%, −79%, and −72%, respectively (Nanzer et al., 2022). The trend remains with a higher AER with increasing BMI (Nanzer et al., 2022). However, it is interesting to note that patients with obesity had a higher reduction in the AER than non-obese patients in the first year but a lower reduction in the second year (Nanzer et al., 2022). In these severe asthma patients, after 2 years, 45%, 25%, and 18% (*p* = 0.007), respectively, remained completely exacerbation-free, with the remainder requiring daily OCS (Nanzer et al., 2022). A significantly higher proportion of non-obese patients remained exacerbation-free which suggests that benralizumab is more effective in non-obese patients for asthma exacerbation than in patients with obesity (Nanzer et al., 2022). This observation could be explained by non-obese asthma patients presenting with less severe asthma at baseline or that patients with obesity experience resistance to this biologic. Interestingly, patients with obesity had the highest ACQ-6 score and lower proportion of controlled asthma than patients with morbid obesity (Nanzer et al., 2022).

While no currently published clinical trials report comparing between mepolizumab, reslizumab, and benralizumab efficacy in obesity, Akenroye *et al* conducted a study in non-obese patients and found minimal significant differences in the efficacy (measured by numbers of exacerbations, FEV1, or ACQ) and safety between mepolizumab, reslizumab, and benralizumab (Akenroye et al., 2022).

### 4.3 Anti-IL-4α

Dupilumab (Dupixent^®^) binds to IL4Rα, blocking IL-4 and IL-13 intracellular signaling, resulting in reduced serum IgE, FeNO, and blood eosinophils (Castro et al., 2020; Ricciardolo et al., 2021c). Dupilumab was FDA-approved in 2017 as a therapy for moderate to severe eosinophilic asthma patients aged ≥12 years with poor control on corticosteroids (DUPIXENT and dupilumab, 2023). This biologic is subcutaneously administered initially at 600 mg (two 300 mg injections) followed by 300 mg/2 weeks or an initial dose of 400 mg (two 200 mg injections) followed by 200 mg/2 weeks (DUPIXENT and dupilumab, 2023). Wenzel *et al* conducted a double-blind placebo-controlled study with 769 severely uncontrolled asthma patients, administering dupilumab 200, 300 mg, or placebo every 2 or 4 weeks over a 24-week period (Wenzel et al., 2016). Wenzel and others noticed that dupilumab improved asthma control, increased FEV1, and reduced severe exacerbations in patients with persistent asthma (Wenzel et al., 2016).

The research on the effects of dupilumab on patients with obesity is limited. Korn *et al* conducted a study aimed to assess the effect of BMI on dupilumab efficacy in uncontrolled severe asthma patients (Korn et al., 2019). The authors conducted a 52-week study where they treated patients in three BMI subgroups (<25 kg/m^2^, 25–29.99 kg/m^2^, ≥30 kg/m^2^) with placebo or the biologic in two dosages (200 mg or 300 mg) (Korn et al., 2019). Using the 200 mg dosage, a −45.7%, −47.9%, and −49.8% reduction in AER was observed for the <25 kg/m^2^, 25–29.99 kg/m^2^, and ≥30 kg/m^2^ BMI categories, respectively (Sposato et al., 2018). In the 300 mg dosage group, a −51.4%, −49.1%, and −35.1% decrease in AER (95% CI) for each BMI category was observed (Korn et al., 2019). Similarly, in the 200 mg dosage group, the authors observed an FEV1 improvement by 57.8%, 49.7%, and 43.7% in each BMI category, respectively, and an FEV1 improvement by 45.6%, 51.4%, and 48.5% was seen in the BMI categories for the 300 mg dosage group (Korn et al., 2019). Across all BMI subgroups, dupilumab reduced severe asthma exacerbations (Korn et al., 2019). Interestingly, 200 mg dupilumab was more effective in reducing asthma exacerbations in the underweight and lean BMI asthma patients while 300 mg was more effective in patients with obesity (Korn et al., 2019). Additionally, in the 200 mg dosage group, patients with obesity had a higher reduction in AER, while in the 300 mg group patients with obesity had the lowest reduction in AER (Korn et al., 2019). In terms of lung function improvement, in the 200 mg dosage group, the greatest observed improvement was seen in underweight asthma patients while in the 300 mg dosage group, the greatest improvement was observed in lean patients (Korn et al., 2019). Although it is established that dupilumab reduces asthma exacerbations, further research is needed to understand the short- and long-term effects of dupilumab on asthma patients with obesity.

Additionally, dupilumab is efficacious and licensed for other type 2-high diseases that frequently coexist with asthma, including atopic dermatitis, chronic rhinosinusitis with nasal polyposis, and eosinophilic esophagitis (Sanofi, 2023). Clinical trials are underway to assess the efficacy of dupilumab in allergic bronchopulmonary aspergillosis and chronic obstructive pulmonary disease (Bhatt et al., 2023). This correlation is only seen in lean patients but further research needs to be done to understand the efficacy of dupilumab in patients with obesity comorbid with these diseases.

### 4.4 Anti-TSLP

Tezepelumab (Tezspire^®^) is a fully human monoclonal IgG2 antibody targeting the alarmin, thymic stromal lymphopoietin (TSLP) (Astrazeneca, 2021; Zoumot et al., 2022). TSLP is produced by lung epithelial cells when dendritic cells are activated in response to allergens, accelerating the differentiation of CD4^+^ native T cells into T2 cells and activation of ILC2s (West et al., 2012; Matsuyama et al., 2022; Parnes et al., 2022). The stimulation of these cells leads to a rapid production of type 2 cytokines such as IL-4, IL-5 and IL-13 which cause allergic reactions (West et al., 2012; Parnes et al., 2022). Studies have shown that the enhanced production of T2 cytokines restimulate T2 skewed cells which contain a much higher level of the TSLP receptor on the cell surface compared to native T cells (Jang et al., 2013). Tezepelumab binds to TSLP, preventing its interaction with its receptor complex predominantly on dendritic cells impairing the downstream activation of signaling pathways such as signal transducer and activator of transcription-3 (STAT3) and Janus kinase-1 (JAK1) that are also involved in T2-low asthma (Menzies-Gow et al., 2021; Corren et al., 2023). Tezepelumab is the first biologic to show a significant impact on severe T2-low asthma (Menzies-Gow et al., 2023). It was FDA approved in 2021 for severe asthma patients (aged >12 years) with uncontrolled symptoms despite corticosteroid therapy (Astrazeneca, 2021). The recommended dosage is 210 mg administered subcutaneously once every 4 weeks (Astrazeneca, 2021). In a double-blind study, Corren *et al* evaluated the efficacy of tezepelumab in 436 patients aged 18–75 years whose asthma remained uncontrolled despite treatment with LABA and ICSs (Corren et al., 2017). The patients received either placebo or tezepelumab, administered subcutaneously, at a dose of 70 or 210 mg/4 weeks, or 280 mg/2 weeks over a 52-week treatment period (Corren et al., 2017). All dosage regimens showed a significant decrease in type 2 biomarkers (eosinophil count, FeNO and serum IgE) and annual asthma exacerbation rates, with the most robust effects being observed with the 210 mg/4 weeks regimen, demonstrating significant anti-inflammatory effects and improvement in lung function compared with placebo (Corren et al., 2017). Interestingly, a similar effect was observed in patients with low FeNO and blood eosinophil levels, suggesting that tezepelumab is clinically relevant in improving airway inflammation in T2-low asthmatics (Dorey-Stein and Shenoy, 2021). Ongoing studies are examining the efficacy of tezepelumab in other severe T2 inflammatory diseases (Dorey-Stein and Shenoy, 2021).

**FIGURE 4:**
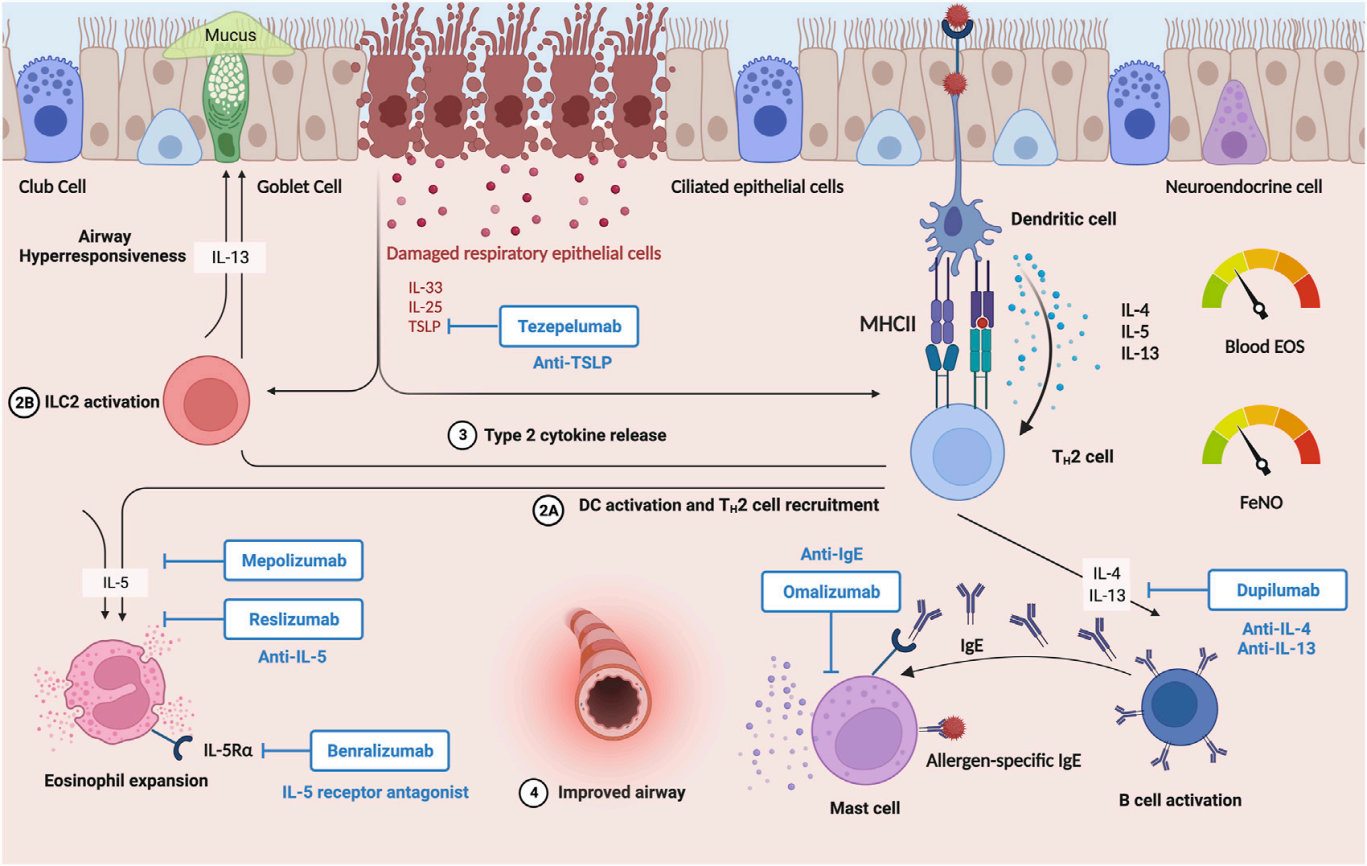
Severe type 2-high asthma patients with comorbid obesity comprise about 44% of asthma patients with obesity (Kaplan et al., 2020). These patients have higher hospital rates, medical costs, and poor control of asthma despite usage of ICS and LABA. Recently, treatment methods for severe asthma improved due to development of biological therapies. The six FDA approved biologics therapies include Omalizumab (anti-IgE); Mepolizumab, Benralizumab, Reslizumab (anti-IL5/anti-IL5R ***α***); Dupilumab (anti-IL4R ***α***); and Tezepelumab (anti-TSLP). These biologics are aimed at various T2 inflammatory pathways to reduce symptoms of asthma, improve lung function, reduce use of oral corticosteroids, and improve quality of life. However, more research needs to be done to see the efficacy of these biologic agents on patients exhibiting asthma and obesity. Created with BioRender.com.

Although no current studies report the effect of tezepelumab in patients with obesity-related asthma, this biologic has promising potential benefits in asthma patients with obesity due its broad effects on both T2-high and -low inflammation. However, additional research is needed to better understand the effects of this targeted biologic treatment on patients with severe, persistent asthma and comorbid obesity (Table 4; Figure 4).

**TABLE 4:** Characteristics of the currently approved monoclonal antibody treatments for severe eosinophilic asthma and reported effects by BMI categories.

Characteristic	Omalizumab	Mepolizumab	Reslizumab	Benralizumab	Dupilumab	Tezepelumab
**Isotype**	IgG1	IgG1	IgG4	IgG1	IgG4	IgG2
**Targeted Epitope**	IgE	IL-5	IL-5	IL-5Rα	IL-4Rα	TSLP
**Drug Administration**	Pre-filled syringe, SC injection	Pre-filled syringe, auto injector pen, SC injection	IV infusion	Pre-filled syringe, auto injector pen, SC injection	Pre-filled syringe, auto injector pen SC injection	Pre-filled syringe, auto injector pen, SC injection
**Patient age**	≥6 years	≥6 years	≥18 years	≥12 years	≥6 years	≥12 years
**Indication**	Severe allergic asthma, childhood asthma	Severe eosinophilic asthma, baseline blood eosinophils ≥150 cells/µL or ≥300 cells/µL in the past year	Moderate to severe eosinophilic asthma, baseline blood eosinophils ≥400 cells/µL	Severe eosinophilic asthma, baseline blood eosinophils ≥300 cells/µL	Moderate to severe eosinophilic asthma, baseline blood eosinophils ≥300 cells/µL	Moderate to severe asthma
**Specific features**	Weight-based dosing	Standard dosage	Weight-based dosing	Administration decreased to every 8 weeks after 3 doses	Weight-based dosing	Effective on T2 high and low asthma
**Key biomarkers for response**	Serum IgE	Elevated blood eosinophils	Elevated blood eosinophils	Elevated blood eosinophils	Elevated blood eosinophils and FeNO	Elevated blood eosinophils and FeNO
**Effect on patients with obesity vs. non-obese BMI**	Contradicting evidence, reduced asthma exacerbations in patients despite BMI	Reduced exacerbations across all BMI categories, possibly less effective with obesity	No studies	Efficacy of benralizumab diminished with increased BMI	Insufficient evidence, possibly more effective in lower BMI	No studies
**Effect on blood eosinophil**	**↓**	**↓↓**	**↓↓**	**↓↓**	**↑** (max increase around 16–20 weeks)	**↓↓**
**Effect on FeNO**	**↓↓**	None	None	None	**↓↓**	**↓↓**
**Effect on serum IgE**	**↓↓**	None	**↓**	**↓**	**↓**	**↓**
**Effect on exacerbations**	**↓↓**	**↓↓**	**↓↓**	**↓↓**	**↓↓**	**↓↓**
**Effect on FEV1**	**↑**	**↑**	**↑↑**	**↑↑**	**↑↑**	**↑↑**
**ACQ-6 score**	**↓↓**	**↓**	**↓↓**	**↓**	**↓**	**↓↓**
**Oral Corticosteroid dependence**	Safely withdrawn between 2 and 4 years after therapy	Yes, about (50%)	Yes, about (50%)	Yes, about (48%)	Yes, about (50%)	Yes, no clear percentage
**References**	[ (Guntern and Eggel, (2020); Owen, (2007); Chipps and Marshik, (2004); Chapman et al., (2006); Kumar and Zito, (2023); U.S. Food and Drug Administration, (2014); Kotoulas et al., (2022); Solèr et al., (2001); Holgate et al., (2009); Xolair, (2023); Niven et al., (2016); Geng et al., (2022); Sposato et al., (2018), Balbino et al., (2020), Drugs.com, (2023)]	[Nucala, (2023), Emma et al., (2018), Albers et al., (2019), Whittington et al., (2017)]	[ Gibson et al., (2016), Pelaia et al., (2019a), Cinqair, (2023), Padilla Galo et al., (2018), Clark and Lebwohl, (2008); Castro et al., (2011); Mukherjee et al., (2018); Farzan et al., (2022), Clinic, (2023)]	[ Pelaia et al., (2018b); Dávila González et al., (2019); Jackson et al., (2020), Trudo and Martin, (2019); Menzella et al., (2021); Akenroye et al., (2022); Nanzer et al., (2022), Liu et al., (2019)]	[ Wenzel et al., (2016); Korn et al., (2019); Castro et al., 2020; Ricciardolo et al., (2021c); DUPIXENT and dupilumab, (2023); Sanofi, (2023), Pharmacoeconomic Review Report, (2018), Mümmler et al., (2021)]	[ West et al., (2012); Jang et al., (2013); Corren et al., (2017); Astrazeneca, (2021); Dorey-Stein and Shenoy, (2021); Menzies-Gow et al., (2021); Matsuyama et al., (2022); Parnes et al., (2022); Zoumot et al., (2022); Corren et al., (2023); Menzies-Gow et al., (2023), TEZSPIRE tezepelumab-ekko, (2023)]

**Abbreviations**: SC, subcutaneous; IV, intravenous; IgE, Immunoglobulin E.

## 5 Future biological therapies

Recent developments in therapeutic strategies and improved understanding of asthma pathogenesis have provided alternatives to corticosteroids as the cornerstone treatment for asthma control. The past two decades have witnessed a heightened development of novel biological anti-cytokine monoclonal antibodies therapies to target eosinophilic inflammation for the management of severe asthma. Even so, new biological agents are being developed especially focusing on non-eosinophilic asthma and alternative, non-T2 inflammatory pathways. While immense improvements in clinical outcomes are observed with the currently FDA approved biologics, a significant disease burden remains from limited understanding of the mechanisms that underly the association between asthma and obesity. While most of the FDA-approved biological therapies target pathways of T2 inflammation downstream of T-helper 2 cell activation, modest asthma control has led researchers to study upstream alarmin cytokines, including IL-25, IL-33, and TSLP. For example, a recent phase II trial showed that Ipetekimab, an anti-IL-33 monoclonal antibody, improved asthma control, lung function, quality of life, and lowered blood eosinophil count (Wechsler et al., 2021). Additionally, in a recent phase IIb randomized control trial (RCT) of patients with moderate-to-severe asthma, amlitelimab, a fully human monoclonal antibody that binds to OX40 receptor and its cognate ligand OX40L, impedes T2 polarization of naive T cells (Lé and Torres, 2022; ClinicalTrials.gov, 2023). In a study done by Arestides *et al*, they found that *Tnfsf4* ^−/−^ ovalbumin-sensitized mice (null for OX40L) had a significant reduction in total serum IgE, pulmonary eosinophils, cytokines, and pulmonary inflammation compared with wild-type control mice (Arestides et al., 2002). Thus, continued research to increase understanding of the type 2 immune response in the airways of asthma patients will reveal promising targets for development of new biologic therapies.

Additionally, alternative methods to deliver biologic therapies are being evaluated. To increase the bioavailability of drugs in the body, researchers are studying nebulized biologic therapy. In a study conducted by Hacha *et al*, the authors evaluated the efficacy of an anti-IL-13 monoclonal antibody in a mouse model of allergic asthma (Hacha et al., 2012). They found that nebulization significantly decreased bronchial hyperresponsiveness and BALF eosinophilia (Hacha et al., 2012). Moreover, inhaled anti-TSLP has been proposed as an alternative administration route. Ecleralimab (CSJ117) represents the first inhaled anti-TSLP antibody that prevents TSLP receptor activation inhibiting downstream inflammatory signaling cascades (Hacha et al., 2012). It is formulated as powder in hard capsules for administration in the airways through a Diskus^®^ (Dry-powder inhaler) (Novartis Pharmaceuticals, 2021). In a Phase IIa trial, ecleralimab reduced allergen-induced bronchoconstriction with mild asthma (NCT04410523) (Novartis Pharmaceuticals, 2021). Nebulizing biological therapies could represent a novel and effective therapy for the treatment of asthma; however, further research needs to be done to investigate the efficacy of these delivery methods in patients with severe uncontrolled asthma.

## 6 Discussion

With rising rates of obesity in the United States, epidemiological data indicates that obesity increases the prevalence and incidence of asthma (Centers for Disease Control and Prevention, 2013). Patients in this subgroup will exhibit more severe, poorly controlled asthma, significantly burdening the healthcare system (Henderson et al., 2020). However, the underlying pathophysiologic mechanisms in which obesity changes asthma are not well understood.

The field of asthma has undergone a radical transformation over the past two decades. Obesity-induced asthma is known to disrupt routine treatments, causing these patients to be resistant to corticosteroids. However, the advent of monoclonal biological therapies has revolutionized the management of severe asthma. Important research has shown the clinical benefits of currently approved monoclonal antibodies on altering levels of biomarkers of type 2 inflammation in lean patients. These patients exhibit significant improvements in asthma exacerbations, lung function, and blood eosinophil count following biologic therapies. Biologics offer a safe, highly effective treatment plan for patients with significant unmet needs through routine treatment. The success of biological therapies deepens our understanding of type 2 airway inflammation and its importance in the pathobiology of asthma.

While biological therapies are an important treatment of asthma patients, none of them are heavily studied or directed specifically toward asthma patients with obesity. Weight-based dosing may improve efficacy of biologics in the population of patients with obesity by optimizing the safety and bioavailability of the drug compared to standard dosing. Research shows that tissue composition is likely to affect drug tissue distribution, absorption, and elimination; therefore weight-based dosing may be beneficial (Cheymol, 1993). Since the roles of IL-4/-13 are pleiotropic with effects on eosinophil transmigrations across endothelium, mucus secretion, B-cell immunoglobulin E production, and enhanced contractility of airway smooth muscle cells, biologic treatment targeting IL-4/-13 may effectively improve asthma in a broader population, not necessarily solely in patients with significant airway eosinophilia (Greenfeder et al., 2001; Castro et al., 2020; Ricciardolo et al., 2021c). Also, in patients with asthma and obesity, anti-IL-5 biologics may have limited effectiveness due to their specific effects on eosinophil maturation, activation, survival, and recruitment to the airway (Padilla Galo et al., 2018; Cinqair, 2023). These drugs to be more effective with weight-based dosing as the effectiveness of IL-5/IL-5R-targeted biologics is maximal in patients with airflow obstruction driven by luminal eosinophils (Pelaia et al., 2019a). Twenty-five percent of non-obese BMI patients with severe T2 asthma are non-responders to the fixed mepolizumab dosage (Özyiğit et al., 2020). Therefore, weight-based dosage may be beneficial as the fixed dose could be under-dosing patients with obesity.

Biologics targeting the alarmins, upstream pathways that initiate events of airway inflammatory and immune responses, have the potential to treat a broader subgroup of patients than the other currently approved biologics. Additionally, it is the only biologic that significantly reduces asthma exacerbations irrespective of key T2 biomarkers and does not target a specific phenotype (eosinophilic or allergic). Findings across clinical trials have shown that tezepelumab inhibits inflammatory biomarkers across diverse pathways causing more effective asthma control (Corren et al., 2017). Since researchers are not fully aware of the cellular and metabolic differences in the airway between lean patients with asthma and patients with asthma and obesity, the ability of tezepelumab to block a broader profile of inflammatory biomarkers may allow for greater reduction in asthma exacerbations in patients with obesity than other biologic therapies. A more precise understanding of patient characteristics and predictive biomarkers could help clinicians decide which biologics lead to the most beneficial response in patients with asthma and obesity. To do so, biomarker responses, asthma control questionnaire scores, and FEV1 should be measured monthly over the course of 2 years to evaluate short- and long-term benefits. These clinical indicators measured by asthma specialists will allow clinicians to execute a course of treatment for this subgroup of patients.

Another concern for biologic treatment of asthma patients with obesity is drug delivery. Efficacy of intramuscularly injected biologic therapies may be compromised in asthma patients with comorbid obesity if needle length is too short to penetrate through excess subcutaneous adipose tissue. Several studies point to significant differences in skin-to-deltoid-muscle distance depending on BMI, sex, injection site and arm circumference (Doppen et al., 2023a; Doppen et al., 2023b). Currently the CDC Guidelines on immunization practices recommend using a 5/8” needle, 23–25 gauge for all patients (Centers for Disease Control and Prevention, 2023). However, use of a standard needle length for all patients does not account for variations in body tissue composition. These differences are crucial because deposition of drug in adipose tissue could result in slower mobilization and processing of the drug, reducing drug bioavailability. In addition, obesity has the potential to affect the rate and extent of drug absorption (Erstad and Barletta, 2022). Further research is needed to evaluate the impact of needle length on efficacy of injected biological drugs in asthma patients with obesity.

While it is clear that the treatment of asthma patients with obesity must include weight control, future research is necessary to fully understand the complex interrelation between obesity and asthma. Many questions remain to be investigated and answered, especially with regard to understanding the efficacy of biological therapies in patient populations with asthma and comorbid obesity (Box 1). More explorations on the metabolic functions of airway cells in asthma patients with obesity, how weight gain affects treatment, and how weight-loss interventions, including medical interventions and bariatric surgery, affect efficacy of therapy are needed. By studying the interaction between the pathogenesis of both disorders, new therapeutic treatments can address the unmet needs of this patient population. Research on asthma and comorbid obesity is especially relevant today and will continue to be in the future as obesity rates are on the rise.

**BOX 1:** Future Areas of Research with Regards to Biologic Therapies in Patients with Asthma and Comorbid Obesity.

Questions for future research
Do the cellular and metabolic functions of airway cells differ between lean patients with asthma and asthma patients with obesity?
Which significant pathways should be targeted by biologics to reach asthma control in patients with obesity? Can serum levels of inflammatory biomarkers predict response to biologic therapy in asthma patients with obesity?
What current biologic treatment is best for patients with asthma and obesity?
What is the average biologic therapy withdrawal time for patients with asthma and obesity? Are the relapse rates significant after withdrawal for biologics in lean patients with asthma *versus* patients with asthma and obesity?
How does the efficacy of biological therapies change through weight loss and gain during treatment?
How does the efficacy of biological therapies change through weight loss with bariatric surgery? How does the efficacy of biological therapies change through pharmacological weight loss (ex: Orlistat)?
Does gender play a role in the efficacy of biologics in patients with asthma and obesity?
Is there a difference in the efficacy of biologics in asthma patients with obesity based on age of onset of asthma or body composition of obesity?
Is standard or weight-based dosing most effective for asthma control in patients with obesity?
Does the mechanism of delivery of the biologic drug affect the bioavailability and clinical symptoms? If so, which delivery method is the best? Does increasing the needle size for injections in patients with obesity increase the effectiveness of the biologic drugs?
Is there a super-responder and/or non-responder population within asthma patients with obesity prescribed biological therapies?
